# Hepatitis A vaccination during ventricular assist device support in heart transplant candidates: immunogenicity and safety in a single-center cohort

**DOI:** 10.1007/s10047-026-01579-2

**Published:** 2026-08-07

**Authors:** Masahiro Arima, Shinya Yamamoto, Maho Adachi-Katayama, Koh Okamoto, Masahiko Ando, Shu Okugawa, Minoru Ono, Takeya Tsutsumi

**Affiliations:** 1 Department of Infectious Diseases, The University of Tokyo Hospital, Tokyo, Japan; 2 Department of Infectious Diseases, Institute of Science Tokyo, Tokyo, Japan; 3 Department of Cardiac Surgery, The University of Tokyo Hospital, Tokyo, Japan; 4 Department of Infectious Diseases, Graduate School of Medicine, The University of Tokyo, 7-3-1 Hongo, Bunkyo-ku, Tokyo, 113-8655 Japan

**Keywords:** Hepatitis A vaccine, Ventricular assist device, Heart transplantation, Artificial heart

## Abstract

Vaccination is among the cornerstones of infectious disease prevention in heart transplant candidates and recipients. Guidelines recommend an inactivated hepatitis A virus vaccine for heart transplant candidates, many of whom are on ventricular assist devices and at risk of hepatitis A virus exposure. However, data on the immunogenicity and safety of hepatitis A virus vaccines in these patients are lacking.This retrospective, observational, single-center study was conducted at our outpatient clinic between December 2019 and December 2025. The study participants were heart transplant candidates with ventricular assist devices aged ≥16 years who tested negative for anti-hepatitis A virus immunoglobulin G at baseline and completed the inactivated hepatitis A virus vaccination series. Anti-hepatitis A virus immunoglobulin G titers were measured after vaccination using a chemiluminescent immunoassay. Thirty-five individuals were included in the study. Immunosuppressive agents were used in four patients (11.4%). All participants achieved seroconversion at a median of 1 month after vaccination. The reverse cumulative distribution of the Anti-hepatitis A virus immunoglobulin G sample/cutoff values indicated that an adequate antibody response was achieved in individuals with ventricular assist devices. Adverse events of the vaccine were minor; two patients (6%) experienced local pain, and two (6%) subcutaneous hemorrhage. Seroconversion in patients with ventricular assist devices was high. hepatitis A virus vaccination should be completed before transplantation in heart transplant candidates, if indicated.

## Introduction

Infectious diseases account for approximately 10% of the causes of death 5 years after heart transplantation [1]. Therefore, prevention measures against infections are crucial for both heart transplant candidates and recipients. In particular, the inactivated hepatitis A virus (HAV) vaccine is one of the cornerstones of infectious disease prevention among solid-organ transplant candidates and recipients and is recommended by several international guidelines [2–4]. The HAV vaccine is also one of the vaccines recommended for individuals who are at risk of HAV exposure because HAV infection severity increases in immunocompromised individuals [3].

However, post-HAV vaccination immunogenicity data of heart transplant candidates are lacking. Although the International Society for Heart and Lung Transplantation (ISHLT) and the American Society of Transplantation (AST) recommend pre-transplant administration of inactivated hepatitis A vaccine for solid-organ transplant candidates [4, 5], these recommendations for heart transplant candidates are largely based on data from other solid-organ transplant populations (e.g., liver and kidney transplantations) or from people with human immunodeficiency virus (HIV) infection. Moreover, pre-transplant vaccines for heart transplant candidates are often administered while candidates are supported with a ventricular assist device (VAD). Because the immunogenicity of HAV vaccines varies widely depending on underlying conditions, data on vaccine responses in heart transplant candidates, particularly those receiving VAD support, are needed. To fill this gap, we aimed to describe our experience on the immunogenicity of an HAV vaccine among heart transplant candidates supported by VAD.

## Materials and methods

### Study design, setting, and patients

This retrospective, observational, single-center study was conducted at the University of Tokyo Hospital, a major heart transplantation center and VAD implantation hospital in Tokyo, Japan. Patients supported only with extracorporeal membrane oxygenation were excluded. All heart transplant candidates on VADs who were evaluated at our outpatient clinic between December 2019 and December 2025, were aged ≥ 16 years, tested negative for anti-HAV IgG at baseline, and received at least two doses of HAV vaccines and subsequently had their antibody titers measured were included in this study. Patients who received one dose, whose post-vaccination anti-HAV IgG level was not measured, or who were referred to other institutions during the HAV vaccination schedule without anti-HAV IgG measurement were excluded.

At our outpatient clinic for infectious diseases in transplant patients, a comprehensive pre-transplant evaluation was provided for all heart transplant candidates. Each candidate’s vaccination and exposure history, along with their serological results, were reviewed. Based on the guidelines recommendations [4], the indicated vaccines are offered and administered to candidates who have agreed to receive them.

Aimmugen^®^ (KM Biologics, Kumamoto, Japan), the HAV vaccine used in Japan, has been approved and is supplied domestically only. It is a freeze-dried, whole-virion, inactivated vaccine that does not contain aluminum. HAV vaccination is highly effective for preventing hepatitis A. In HIV-negative healthy individuals, Aimmugen^®^ achieves 85% anti-HAV immunoglobulin (Ig)G seropositivity after the second dose [6]. The interval between the first and second doses was 4 weeks. The third dose (booster) was administered at least 24 weeks after the first dose according to the manufacturer’s instructions. After the last dose, anti-HAV IgG levels were measured using a chemiluminescent immunoassay (HAVAB-G ABBOTT, ABBOTT JAPAN, LSI Medience Corporation, Tokyo, Japan). A patient was considered positive for anti-HAV IgG when the titer was > 1.0 (sample/cutoff [S/CO]). The S/CO value can range from 0 to 998,000. Seroconversion was confirmed if anti-HAV IgG positivity was achieved at any point after vaccination.

### Data collection and analysis

 The patients’ electronic medical records were manually reviewed. Using a standardized data collection form, information on the following variables was collected at the time of the first HAV vaccine dose: age, sex, body mass index, smoking history, Charlson Comorbidity Index, underlying etiology of heart failure, history of previous transplantation, receipt of hemodialysis, use of immunosuppressants, New York Heart Association classification, laboratory data, anti-HAV IgG results, vaccine-related adverse events, and number of vaccine doses. We collected HAV-IgG titers and calculated their reverse cumulative distribution. We classified responses with S/CO ≥ 10 as strongly reactive, following a prior study [7]. All statistical analyses were performed using JMP Pro (version 17; SAS Institute, Tokyo, Japan), Statistical Package for the Social Sciences (Chicago, IL, USA), and GraphPad Prism 9.0 software (GraphPad Software, San Diego, CA, USA).

**Fig. 1:**
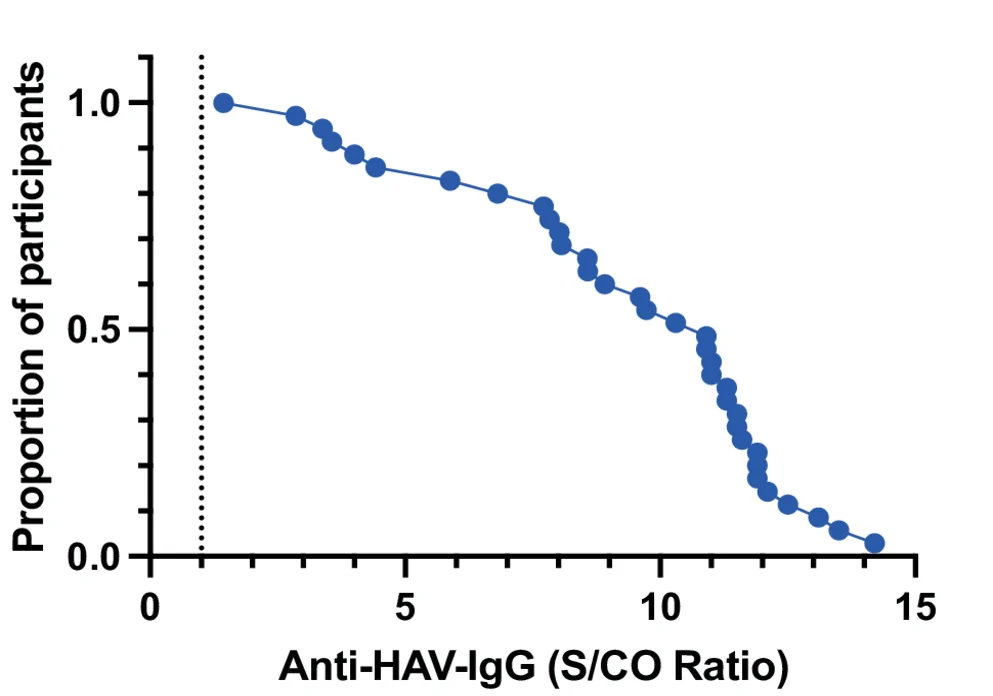
Reverse cumulative distribution curves of anti-HAV IgG titers. The blue dots indicate HAV IgG titers (S/CO ratio), and the scores are plotted on the x-axis. The cut-off value for HAV IgG positivity is 1 S/CO. HAV: hepatitis A virus; Ig: immunoglobulin

**Table 1:** Baseline clinical characteristics and outcomes in hepatitis A vaccine recipients

Characteristics	
Age (years)	43 (37–50)
Sex (female, %)	15 (42.8%)
Body mass index (kg/m^2)	22 (19–23.4)
Past medical history	
Dilated cardiomyopathy	17 (48.5%)
Hypertrophic cardiomyopathy	3 (8.5%)
NYHA functional classification	
I	19 (54.3%)
II	9 (25.7%)
III	2 (5.7%)
IV	1 (2.9%)
Smoking history	11 (34%)
Hemodialysis	0
Prior transplantation history	0
Charlson comorbidity index (score)	2 (1–2)
Laboratory data	
WBC count (count/L)	6,400 (5,000–7,300)
Lymphocytes (count/L)	1200 (800–1700)
Hemoglobin (g/dL)	13 (12.2–14.7)
Albumin (g/dL)	4.3 (4.2–4.6)
Creatinine (mg/dL)	0.84 (0.74–0.93)
Immunosuppressive agents	
Systemic corticosteroids	3 (8.5%)
Steroid enemas	1 (2.9%)
Side effects from vaccine	
Pain	2 (5.7%)
Subcutaneous hemorrhage on vaccine site	2 (5.7%)
Outcome	
Seroconversion (HAV-IgG positivity after vaccination)	35 (100%)

Values are indicated as median (interquartile range) or number (%).

NYHA: New York Heart Association, WBC: white blood cells, HAV: hepatitis A virus; Ig: immunoglobulin.

## Ethical approval

All procedures were performed in accordance with institutional guidelines and the Declaration of Helsinki. This study was approved by the institutional review board of the University of Tokyo Hospital (approval number 2025274NI). Given the retrospective nature of the study, the requirement for informed consent was waived by the board. This study was reported in accordance with the Strengthening the Reporting of Observational Studies in Epidemiology guidelines [8].

## Results

A total of 48 patients were evaluated at our clinic during the study period. Of these, 35 (73%) met the inclusion criteria. The reasons for exclusion were the lack of an anti-HAV IgG test after vaccination (*n* = 8), incomplete schedule due to heart transplant (*n* = 4), and transfer to another hospital (*n* = 1). The baseline characteristics of the patients are summarized in Table 1. The median age was 43 years (interquartile range [IQR], 37–50 years), and 15 patients (42.8%) were female. The median body mass index was 22 (IQR, 19.0-23.4). Dilated cardiomyopathy was the most common underlying etiology (17/35, 48.5%), followed by hypertrophic cardiomyopathy (3/35, 8.5%). The median New York Heart Association class and Charlson comorbidity index were 1 (IQR, 1–2) and 2 (IQR, 1–2), respectively. None of the patients was on hemodialysis or had a history of transplantation. Three patients (8.5%) had received low-dose systemic prednisolone (5 mg daily). Laboratory findings are also summarized in Table 1. No remarkable abnormalities were identified.

HAV IgG levels were measured at a median of 1 month after vaccination. Overall, 100% (35/35) of the patients achieved seroconversion. The RCD of anti-HAV-IgG S/CO values is shown in Fig. 1. 50% of the RCD was above 10 S/CO indicating strong reactivity has been achieved in VAD-supported patients in this cohort. The anti-HAV IgG S/CO of four patients treated with immunosuppressive agents were 8.0-11.3. The adverse events associated with the vaccine were minor; two (6%) patients reported local pain, and two (6%) had subcutaneous hemorrhage.

## Discussion

This retrospective study was conducted among VAD-supported heart transplant candidates to elucidate their HAV vaccination status and related clinical findings. Thirty-five VAD-supported heart transplant candidates completed a series of the inactivated HAV vaccine, and all showed seroconversion. Moreover, the RCD of anti-HAV IgG values indicated adequate antibody responses across the cohorts, and the adverse events were minor.

In general, vaccine responses, including those to the HAV vaccine, can be attenuated by immunosuppressive conditions. There is only one previous study addressing the immunogenicity of a vaccine among VAD-supported patients. A prospective single-center study on the immunogenicity of the COVID-19 mRNA vaccine showed that 45% of heart transplant recipients and 83% of VAD-supported patients had seroconversion of anti-spike antibody [9]. No studies on the immunogenicity of HAV vaccines among patients with advanced heart failure without VAD have been reported. Among solid organ transplant candidates, previous studies have shown that the immunogenicity of HAV vaccines is 97 − 100% among patients on hemodialysis, and 26–66% among those with advanced cirrhosis [10–13]. While no data on heart transplant recipients has been reported, previous studies have shown that 27–72% of kidney transplant recipients and 23–97% of liver transplant recipients achieve seroconversion with HAV vaccines [14–17].

Moreover, we found that half of the participants had an S/CO value exceeding 10. Although the S/CO value is not a quantitative measure and cross-assay comparisons cannot be performed, this finding, together with the observed seroconversion rate, suggests that patients receiving VAD support can mount a reasonable immune response to HAV vaccination. Taken together, our findings further contribute to the existing literature supporting the recommendation to administer HAV vaccines to patients supported by a VAD before heart transplant, if indicated.

The clinical relevance of these findings may vary according to national hepatitis A vaccination policies. In the United States, universal routine hepatitis A vaccination of children was introduced in 2006 [18], whereas vaccination policies vary across European countries. In Japan, hepatitis A vaccination is not included in the routine childhood immunization program. Consequently, many adult heart transplant candidates, including those receiving VAD support, may remain susceptible to HAV infection and should be assessed for the need for hepatitis A vaccination before transplantation.

This study has some limitations. The study could not fully evaluate the antibody response changes over time, such as the time to transplantation, and post-transplant kinetics. Almost all anti-HAV IgG S/CO values were evaluated 1 month after the last HAV vaccination, which is associated with high titers. Future longitudinal studies should examine the changes in S/CO value distribution over time and the persistence of antibody responses after the initiation of immunosuppression. These include the use of a single HAV vaccine formulation and the low prevalence of non-cardiac comorbidities that could impact vaccine response.

## Conclusion

In conclusion, seroconversion after inactivated hepatitis A vaccination was high among heart transplant candidates supported by ventricular assist devices, and vaccine-related adverse events were minor. Hepatitis A vaccination should be completed before transplantation when indicated.

## Data Availability

This study does not report any original code. Any additional information required to re-analyze the data reported in this paper is available from the corresponding author upon request.

## Declarations

### Research field

Artificial Heart (clinical).
